# When Priests Become Predators: Profiles of Childhood Sexual Abuse Survivors

**DOI:** 10.3402/qhw.v8i0.20937

**Published:** 2013-06-18

**Authors:** Patricia Wolskee

Prior to the writing of this book, “**When Priests Become Predators,”** and prior to 2002, few researchers in psychology had empirically investigated the problem of sexual abuse, specifically by clergy. Prior work was based on very small sample sizes involving perhaps one or two diocese. The year 2002 was a watershed year in that abuse carried out by Boston theologians was made public resulting in awareness of the Catholic Church's contribution to allowing these religious priests to continue their abusive behavior by transferring them to other areas and turning a blind eye to the atrocities they continued to commit. While this story was widely reported in the press, journalists are limited by space and the language that can be used. “**When Priests Become Predators**” by Tom Neuberger presents ten individual cases and gives the survivors a voice to be heard by others, who may or may not wish to hear their testimonies. The accounts given are brutally honest, graphic and focused. The book goes far and beyond any prior attempt to expose the injustices and ruined lives of these ten survivors.

The book is divided into two sections, the first presents the stories of those who survived sexual abuse by priests in the state of Delaware, while the second half covers the trial of John Michael Vai. The latter section contains his courtroom testimony, as a juror would hear it. As such, the text is unedited and the language used is blunt and truthful, making it difficult to read for many reasons.

What can be done now to support these survivors and the hundreds of thousands more who suffer alone in fear of revealing themselves as they seek help? As Tom Neuberger argues, this book can be a contribution to other professions and I propose that it can be used as an adjunct text to help those working and teaching in the field of psychology. It presents the actual phenomenological experiences of child abuse. Other studies in the field of psychology have used or attempted to use quantitative methods to study sexual abuse by priests and the resultant effect on the survivors. This book is an in depth phenomenological study of ten stories recounted by ten people. Moustakas (1994), one of the fathers of qualitative inquiry, has used phenomenological data analysis in his modified Stevick-Colaizzi-Keen method of research. Not only is this book a qualitative gold mine but it is also a verbatim account of many survivors’ stories. One use of the book in psychology today could be as a tool to illustrate the application of qualitative methodology and how phenomenological material can be dissected for analysis. This aspect alone warrants the use of this book in psychological research. A second use is to expose and raise awareness of how offenders and victims think. Neuberger's work offers a rare opportunity to access this in depth and with credibility, as he presents the sworn courthouse testimony of the survivors. Although a significant volume of research exists on sex offenders, little deals specifically with abuse carried out by priests using such rich phenomenological data. Other research areas that would find the book useful include child development, cognitive psychology, health psychology, neuropsychology and others.

Tom Neuberger has achieved something new with his work. He was able to find these survivors and then interview and defend them against their predators. His efforts resulted in large settlements. However, these settlements will not bring back the survivors’ lost years of innocent childhood. Their adult lives have also been altered due to the sexual abuse and related effects, including posttraumatic stress disorder (PTSD, DSM-IV TR code 309.81). PTSD has become almost a household word now after the attention it received in relation to the returning Iraqi war veterans. The disorder is now better understood in the realm of psychology and better understood by trauma survivors. (i.e. car accident victims and spouse who were abused).

The stories of the survivors discussed in this book allow one to see and understand what posttraumatic stress disorder is and how it can change the life of an individual who has been exposed to the horrors of sexual abuse by those they trusted (i.e. family members, neighbors, friends and the local Catholic priest). Research shows that these men are no different from other sex abusers (Dale & Alpert, 2007). They took the lives of these young boys and turned them into a string of endless anxiety. For example, as boys they spent time worrying that a certain priest who made a regular point of needing to speak to them for different reasons would approach them. During the 1950s to 1980s priests had a regular base from which to choose young boys to sexually abuse under the guise of the “cloth” and cover of the Catholic Church. Abusers were found among the newest priests to the bishops and cardinals, all of whom took advantage of the power and control they maintained. This aspect of control is discussed in the text by one of the survivors, Douglas McClure, Sgt. U.S.M.C. His own description of how this affected his life can be read and felt in the book. He lost his sense of self and own personal identity as discussed clinically by Farrell (2009). He stated, “Many struggle with the unbearable conviction that they are fated to live ‘in the skin’ of an identity that is not an authentic experience of the person they were meant to be.” (p. 2).

In sum, as a psychologist I find this book to be highly useful in a teaching, clinical or counseling environment. It offers in depth knowledge of a difficult topic and real world experience through the personal stories of others. Its content is credible; it is derived from legal records and was compiled by a legal representative who checked every detail. It offers a unique opportunity to study these survivors and many others. No pseudo cases need to be presented to illustrate the thoughts and ideas of survivors for those in academia; the stories speak for themselves. That, in and of itself, makes this book, “**When** P**riests Become Predators,”** an outstanding tool for the study of the sexually abused. This book should have a place on the bookshelf of any psychologist as well as those working with survivors of sex abuse, those suffering with PTSD, those wanting more knowledge and those who truly want to be informed in this area. We have Tom Neuberger to thank for this rare opportunity.

## Author of this book review:

Patricia Wolskee, PhD who has been a health psychologist for 30 years with a focus on chronic pain management and other health challenges. She has published numerous works both nationally and internationally. She presently continues her work in health psychology as adjunct professor, Northcentral University in Prescott AZ.
